# Phenotypic Characterization, Genetic Diversity Assessment in 6,778 Accessions of Barley (*Hordeum vulgare* L. ssp. *vulgare*) Germplasm Conserved in National Genebank of India and Development of a Core Set

**DOI:** 10.3389/fpls.2022.771920

**Published:** 2022-02-24

**Authors:** Vikender Kaur, J. Aravind, Manju, Sherry R. Jacob, Jyoti Kumari, Bhopal S. Panwar, Narendra Pal, Jai C. Rana, Anjula Pandey, Ashok Kumar

**Affiliations:** ^1^National Bureau of Plant Genetic Resources (ICAR), New Delhi, India; ^2^Department of Botany, Baba Mast Nath University, Rohtak, India; ^3^Bioversity International, National Agricultural Science Complex (NASC), New Delhi, India

**Keywords:** barley germplasm, characterization, core set, genebank, genetic diversity, *Hordeum vulgare*, India

## Abstract

The entire collection of cultivated barley germplasm accessions conserved in the Indian National Genebank (INGB) was characterized for nine qualitative and 8 quantitative traits to assess the nature and magnitude of prevailing genetic variability and to develop a core set. A wide range of variability was observed for days to spike emergence (51–139 days), days to physiological maturity (100–152 days), plant height (45.96–171.32 cm), spike length (3.44–13.73 cm), grain number/spike (10.48–82.35), and 100-grain weight (1.20–6.86 g). Initially, seven independent core sets were derived using 3 core construction tools– MSTRAT, PowerCore, and Core Hunter 3 by employing the maximization method, heuristic sampling, and optimisation of average genetic distances, respectively. The core set-3 generated by Core Hunter 3 by simultaneous optimisation of diversity and representativeness, captured maximum genetic diversity of the whole collection as evident from the desirable genetic distance, variance difference percentage (VD; 87.5%), coincidence rate of range (CR; 94.27%) and variable rate of coefficient of variance (VR; 113.8%), which were more than threshold value of VD (80%), CR (80%), and VR (100%) required for good core collection. The coefficient of variation and Shannon–Weaver diversity indices were increased in the core set as compared with the whole collection. The low value of Kullback-Leibler distance (0.024–0.071) for all traits and quantile-quantile plots revealed a negligible difference between trait distribution patterns among the core set and entire assembly. Correlogram revealed that trait associations and their magnitude were conserved for most of the traits after sampling of the core set. The extraction of the INGB barley core set and identification of promising accessions for agronomically important traits in different genetic backgrounds will pave the way for expedited access to genetically diverse and agronomically important germplasm for barley breeding.

## Introduction

Barley is one of the main important cereal crops cultivated worldwide in a wide range of environments from temperate to sub-tropical, arid to semi-arid. Ranking fourth in world cereal production after maize, rice, and wheat, it is mainly used for feed (55–60%), brewing malts (30–40%), and the remaining percent for food purposes (Ullrich, 2010; Kumar et al., 2014). Cultivated barley (*Hordeum vulgare* ssp. *vulgare*) evolved from its wild progenitor *H. spontaneum* (C. Koch) Thell, in the Fertile Crescent of Middle East about 10,000 years ago (Harlan and Zohary, 1966; Ceccarelli et al., 1999; Badr et al., 2000) and has different morphological variants based on inflorescence and caryopsis types. They are classified into two distinct morphotypes based on the number of grain rows in a spike *viz*. two-rowed (*H. vulgare* ssp. *distichum*) and six-rowed (*H. vulgare* ssp. *hexastichum*). In two-rowed barley, only one spikelet at each node is fertile while all three spikelets are fertile in the six-rowed variant. Another classification is based on the presence of distinct caryopsis types. Most cultivars have intact husk cover of caryopsis (hulled), wherein the outer lemma and inner palea are firmly adherent to the pericarp at maturity, but a few variants are of the free threshing type (with a loose husk cover which gets easily separated from grain upon threshing) and are called naked or hulless barley (*H. vulgare* var. *nudum* L.) (Manjunatha et al., 2011). In addition, colored caryopses are also reported in both hulled and hulless types.

These distinct morphotypes have specific agronomic value and are utilized for different purposes. The six-rowed form is cultivated worldwide for feed purposes under low input, rain-fed conditions, and two-rowed is generally grown for malt purposes under high-input agriculture systems. Naked barley is a staple food crop of people residing in high-altitude areas where no other crop can be successfully grown and is relished in the form of “sattu.” It is cultivated in altitudes up to 4,400 m above mean sea level in Nepal, India, Bhutan, Tibet, China, Korea, Japan, and Ethiopia for human consumption (Sun and Wang, 1999; Assefa and Labuschagne, 2004; Manjunatha et al., 2007). Until the late nineteenth century, all barleys existed as highly heterogeneous populations with no crossing barrier between the wild *spontaneum* and cultivated forms (Asfaw and von Bothmer, 1990), thus spontaneous crossing led to wide genetic variation and a high degree of natural tolerance in barley to major abiotic stresses such as drought and salinity (Zhao et al., 2010; Qiu et al., 2011; Allel et al., 2016). During the process of domestication, gradual selection was attempted to accumulate six-rowed spikes, naked caryopsis, and non-brittle rachis as the key traits to facilitate agricultural production (Salamini et al., 2002). Modern agriculture led to the replacement of landraces and diverse germplasm by pure line varieties resulting in more vulnerability to climate change episodes and biotic and abiotic stresses. Therefore, an extensive characterization of prevalent diversity among the genebank collection of barley germplasm is required to diversify parental material for the breeding and development of an effective crop improvement program. Agro-morphological characterization has long been designated as the foremost important step to assess genetic variability, to discriminate among materials from different geographic sites, establish core collections and prioritize accessions for effective use in breeding. Many studies on barley assemblies across different regions of the world *viz*. Spain (Lasa et al., 2001), North America (Mikel and Kolb, 2008; Allel et al., 2017), Jordan (Shakhatreh et al., 2010), Nordic countries (Bengtsson et al., 2017), Ethiopia (Abebe et al., 2008, 2010), Slovakia (Žáková and Benková, 2004), Bhutan (Konishi et al., 1993), and India (Manjunatha et al., 2007; Sarkar et al., 2010; Kaur et al., 2018; Manju et al., 2019) have highlighted the importance of phenotypic characterization.

The large size and heterogeneity of germplasm collections conserved at genebanks complicates the characterization, evaluation, utilization, and maintenance of these resources. To increase the efficiency of characterization and utilization of these genebank collections, the concept of “Core collections” was introduced while preserving the maximum genetic diversity of the entire collection (Frankel, 1984; Brown, 1989). The smaller size of a core collection allows efficient management, characterization, and evaluation of genetic resources and is cost-effective. Until now, few efforts to consolidate barley germplasm resources into core collections have been done. The National Small Grains Collection (NSGC) barley core was established in 1995 (with final additions in 2006) using approximately 10% of the entire collection of 33,176 accessions of the USDA-ARS (NSGC) barley germplasm collected from more than 100 countries (Bockelman and Valkoun, 2011; Bonman et al., 2011). This core set was constituted by randomly selecting the accessions based on the logarithm of the total number of entries from each country and with a minimum of one accession per country (Bockelman and Valkoun, 2011). The international Barley Core Collection (BCC) was constituted by a set of 1,600 accessions encompassing diverse material representing cultivated and wild species of *Hordeum* from *ex-situ* collections around the world and is maintained by a number of genebanks globally (Knüpffer and van Hintum, 1995, 2003). Another is the Spanish barley core collection with a total of 160 entries constituted by old varieties (15), entries in common with previously existing barley core collections (15), 2-row (8), and 6-row (122) entries extracted from a collection of about 2,000 barley accessions conserved at Spanish National Germplasm Bank (Banco Nacional de Germoplasma, BNG) (Igartua et al., 1998). A molecular core set of 1,000 accessions was extracted by analyzing the genotyping-by-sequencing (GBS) data from a total of 22,626 DNA samples covering the entire barley collection of the German federal *ex situ* genebank hosted at the Leibniz Institute of Plant Genetics and Crop Plant Research (IPK), Gatersleben (Milner et al., 2019). The data on phenotypic traits and single nucleotide polymorphism (SNP) profiles is accessible for exploration through a web application tool, BRIDGE (König et al., 2020).

The Indian National Genebank (INGB) at the Indian Council of Agricultural Research-National Bureau of Plant Genetic Resources (ICAR-NBPGR), New Delhi, conserves the base collection with about 7,500 accessions of barley which were characterized for this study. After removing the duplicates and accessions in which data could not be recorded for one or more traits, finally, 6,778 accessions were subjected to data analysis to assess genetic diversity and develop a core set in barley. The objective of this work was to assess the nature and magnitude of prevailing diversity and to provide a manageable and representative selection in the form of a core set for use in research and breeding. Initially, seven independent core sets were assembled using different core construction softwares- MSTRAT, PowerCore, and Core Hunter 3 (CH3) which were subjected to quality evaluation based on different criteria (Odong et al., 2013) to select the most desirable core set representing maximum diversity/richness and representativeness of full range of variation present in the whole collection. The present research report describes the first systematic large-scale phenotypic characterization of barely accessions predominantly of Indian origin held in INGB and the development of a multipurpose core collection maximizing both representativeness as well as diversity simultaneously. In addition, the identification of trait-specific diverse accessions will facilitate effective utilization of prevalent diversity in barley improvement.

## Materials and Methods

### Plant Materials and Field Experiment

**Figure 1:**
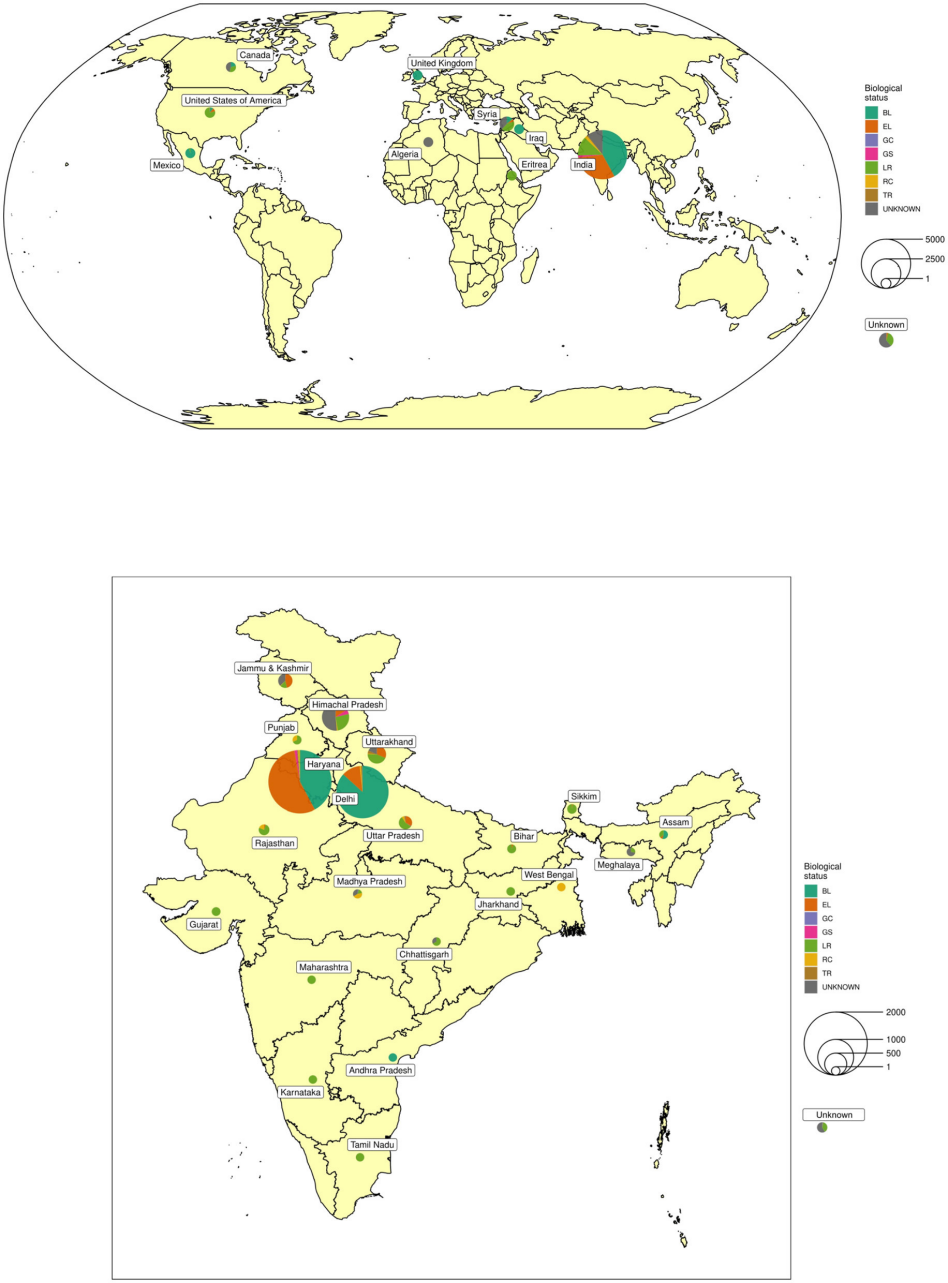
Geographical distribution of collection sites and biological status of exotic and indigenous barley conserved at National Genebank of India.

A total of 6,778 barley accessions, of which 5,351 were indigenous collections (IC) assembled from 21 different states of India and 1,427 exotic collections (EC) mainly from Syria, Canada, the USA, and Mexico were used in this study (Figure 1 and Supplementary Table 1). The accessions were sown from 18th to 23rd November 2016 and from 16th to 20th November 2017 at ICAR-NBPGR, experimental farm, Issapur, New Delhi located at a latitude of 28°57′ N, longitude of 76°84′ E, and altitude of 218 m above mean sea level. The farm has a semi-arid subtropical climate with average annual precipitation of 400 mm. The soil of the farm varies from sandy loam to loamy sand with a pH of around 8. Each accession was grown in two rows of 2 m length, with a between-row spacing of 45 cm, and within-row spacing of 10 cm in an Augmented Block Design (Federer, 1956) with 40 blocks. Each block had 170 accessions while the last block was incomplete. Five check varieties representing six-rowed hulled barley: BH902, BH959, RD2552; hulless: BHS352, and two-rowed: DWRB101 were replicated randomly in each block. The crop was fertilized with 60:30 kg/ha of N:P_2_O_5_ with entire P along with half of N fertilizer at the time of sowing, while the remaining half N was top-dressed after first irrigation. Besides pre-sowing irrigation for crop establishment, first irrigation was done at active tillering (30–35 DAS) and 2nd at flowering stage (65–70 DAS). Recommended agronomic practices were followed during various stages of crop growth. Each accession was harvested and threshed manually. Additionally, a selected set of around 206 diverse accessions showing superiority for different agronomic and phenological traits was further validated in the subsequent years (2018–19 and 2019–20) in an Augmented Block Design with 40 entries in the first 5 blocks and 42 entries in 6th block including the above-mentioned checks.

### Phenotyping of Agro-Morphological and Phenological Traits

The data were recorded on 17 traits, of which 8 traits *viz*. days to 75% spike emergence (DSE), days to 80% maturity (DPM), plant height (PH, cm), spike length (SL, cm), spikelet triplet groups per spike (STG), grain number per spike (GNS), hundred-grain weight (HGW; g), and grain yield per meter row (GY, g) were measured quantitatively, while another 9 traits *viz*. growth class (GC), growth habit (GH), early plant vigor (EPV), spike row type (SR), awn type (AT), spike density (SD), grain type (GT), grain pericarp color (GPC) and lodging tendency (LT) were assessed qualitatively as per descriptors for barley (Mahajan et al., 2000). Herbarium specimens of distinct types were deposited in the National Herbarium of Cultivated Plants (NHCP) at NBPGR under the number (HS23243–HS23251).

### Statistical Analysis

The qualitative trait data were subjected to Analysis of Variances (ANOVA) according to Augmented Randomised Complete Block Design (Federer, 1956) and adjusted means were generated in R R Core Team (2019) with the package augmentedRCBD (Aravind et al., 2019) which were then utilized for further analysis. Genetic variability parameters such as phenotypic coefficient of variation (PCV) and genotypic coefficient of variation (GCV) were computed for each trait (Burton, 1952) and the range was categorized as per Sivasubramanian and Madhavamenon (1978). Estimation of broad-sense heritability was done as $h_{\left(bs\right)}^{2}=\;\frac{V_{G}}{V_{P}}$ (Lush, 1940), where *V*_*G*_ and *V*_*P*_ are the genotypic and phenotypic variances respectively and it was further classified into low, medium, and high as per Robinson (1966). Expected genetic advance (EGA) was calculated using the formula $EGA=k\times \sqrt{V_{G}}\times h_{\left(bs\right)}^{2}$ as per Johnson et al. (1955), where *k* is the standardized selection differential whose value is 2.06 at 5% selection intensity. Genetic advance expressed as a percentage of mean was calculated as $GA\left(\%\right)=\frac{EGA}{mean}\times \;100$.

### Development of Core Set

Compiled data on agro-morphological and phenological traits were standardized to eliminate scale differences and employed in core set identification. Initially, seven different core sets were developed which consisted of one core set developed using MSTRAT (Gouesnard et al., 2001) through the maximization method, another core set by heuristic method with PowerCore (Kim K. W. et al., 2007), and five core sets using the R front-end for Core Hunter 3 (CH3) software (De Beukelaer and Davenport, 2018; De Beukelaer et al., 2018) following optimisation of average genetic distance-based criteria as described in Odong et al. (2013). Five different strategies based on the optimisation of average genetic distances between each accession and nearest entry in the core (A-NE) and the average distance between each entry and nearest neighboring entry (E-NE) were employed measuring Gower distance. These include:

(i.) maximizing E-NE distances (CH3- Core set 1);
(ii.) maximizing A-NE distance (CH3- Core set 2);
(iii.) maximizing both E-NE and A-NE with equal weightage of 1:1 (CH3- Core set 3);
(iv.) E-NE and A-NE with unequal weightage of 0.3:0.7 (CH3- Core set 4);
(v.) E-NE and A-NE with equal weightage of 0.7:0.3 (CH3- Core set 5).

A set of selected 83 accessions was fixed for inclusion in all core sets based on the superior expression for one or more traits visualized in the field, trait-specific promising accessions, and limited representation in the genebank. The size of the core set was fixed as approximately 10% of the entire collection, considering the neutral allele theory (Brown, 1989).

### Evaluation of the Core Set

All of the seven core sets were compared according to the genetic distance criteria as described in Odong et al. (2013), as well as other statistical parameters such as mean difference percentage (MD%), variance difference percentage (VD%), variable rate of coefficient of variance (VR%), coincidence rate of range (CR%) for quantitative traits as per Hu et al. (2000), and the “coverage” criteria for qualitative traits (Kim M. J. et al., 2007). In addition, the Mantel test (Mantel, 1967) was performed to compute the correlation between two trait correlation matrices i.e., each core set and the entire collection. After the selection of desired core set showing maximum diversity and representativeness, further comparative analysis of quantitative trait means of the selected core set and the entire collection was done by Newman-Keuls test (Newman, 1939; Keuls, 1952) and *t*-test. For quantitative traits, the homogeneity of variances of the entire collection and the selected core set was examined with Levene's test (Levene, 1960), and the difference of frequency distribution was evaluated by the Wilcoxon rank test (Wilcoxon, 1945). Boxplots were drawn to determine and compare the frequency distribution among the whole collection and core set. The quantile-quantile (QQ) plots (Wilk and Gnanadesikan, 1968) were plotted for an overall visual view of distribution difference of continuous traits between core set and entire collection and Kullback-Leibler distance (Kullback and Leibler, 1951) was calculated. The Shannon-Weaver diversity index (H') and evenness were computed using the phenotypic frequencies of qualitative characters (Shannon and Weaver, 1949). The Pearson correlation coefficients were computed to assess the interrelations between different quantitative and qualitative traits in both the whole collection and core set. Principal Component Analysis (PCA) was employed to identify the relationships between different traits and their contribution to multivariate variation. All the statistical evaluations of the core sets were done using the R package EvaluateCore (Aravind et al., 2020).

## Results

### Variability for Qualitative Traits

**Figure 2:**
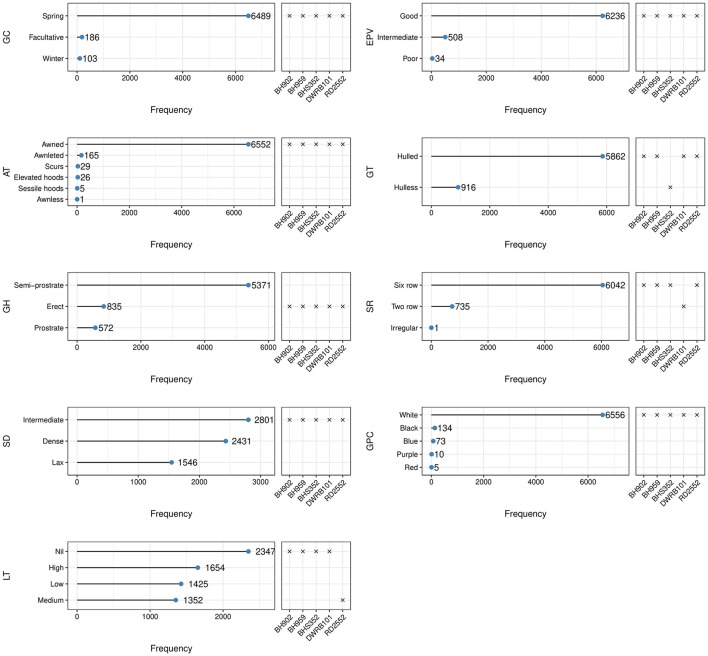
Frequency dot plots of 6,778 barley accessions for qualitative traits.

**Figure 3:**
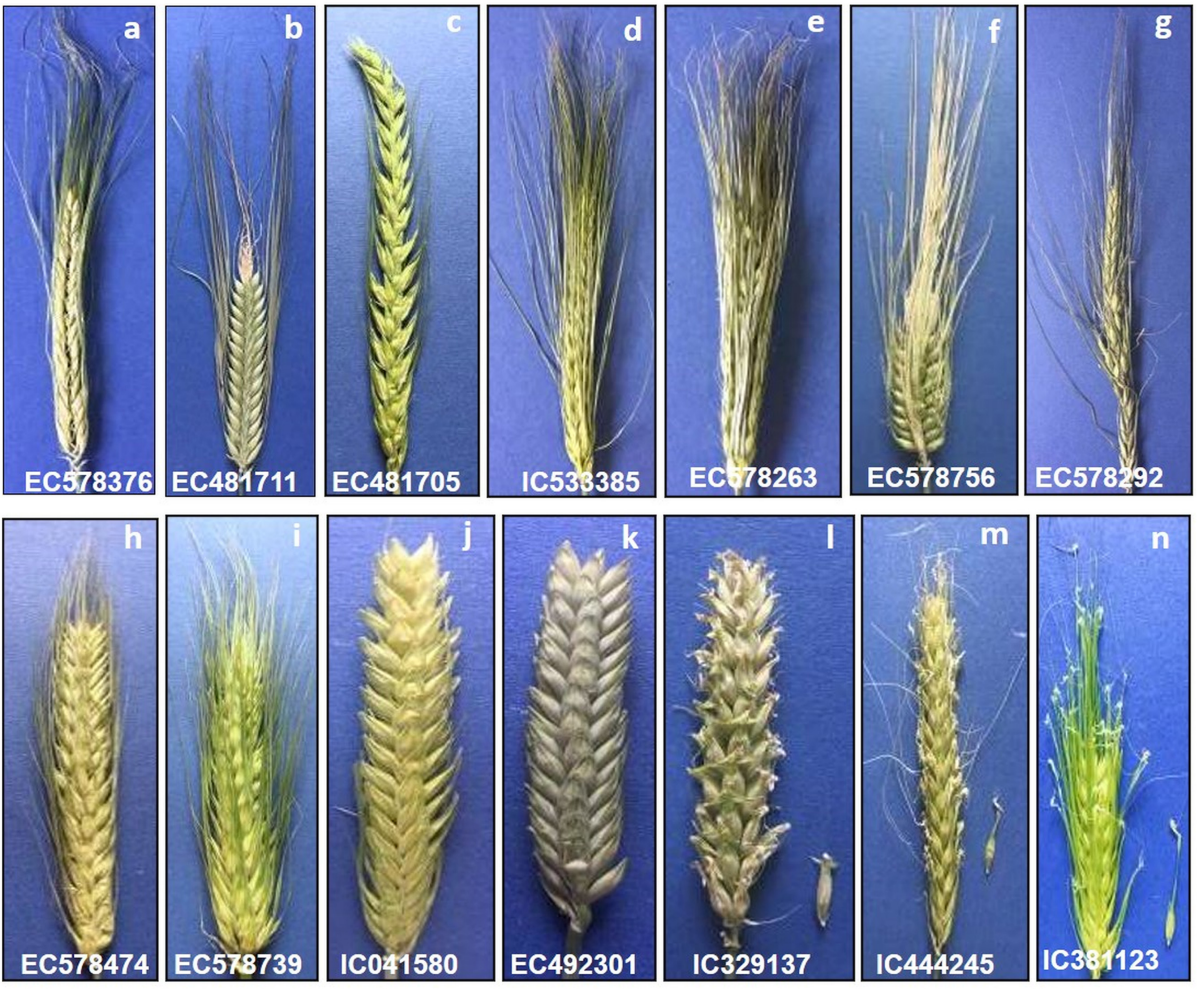
Variability in spike shape, density, and awn types in barley germplasm–**(a)** Two-rowed oblong spike with long awns; **(b)** Two-rowed fusiform spike; **(c)** Lax two-rowed fusiform spike showing the presence of scurs; **(d,e)** Six-rowed oblong spike with long awns; **(f)** Elliptical six-rowed dense spike; **(g)** Irregular spike showing the formation of additional florets; **(h)** Scurs in central and lateral rows all over the spike; **(i)** Awnleted elliptical spike; **(j)** Scurs in central and basal rows only; **(k)** Awnless spike; **(l)** Sessile hoods; **(m)** Elevated hoods with projection at small height; **(n)** Elevated hoods with projection at more height.

Traits measured qualitatively showed a wide range of variation (Figure 2). Most of the accessions (96%) under study were of spring barley, while winter and facultative barley were very few, which were mainly from the exotic collections. Early plant vigor, which serves as the proxy for the subsequent growth of the plant, was good for 92% of the accessions. Semi-spreading/semi-prostrate growth habit was recorded for 79% accessions while 12% were erect and 9% were of spreading/prostrate type. The spike structure was characterized as six-rowed in 89% accessions and two-rowed in 11% accessions. The spike density was recorded as dense (rachis internode length <2 mm) in 36%, lax (rachis internode length >4 mm) in 23%, and intermediate with rachis internode length between 2 and 4 mm in the remaining accessions. Around 35% of accessions showed no lodging, 24% showed high lodging, and 41% were recorded with low to medium lodging tendency. Around 13% (916 accessions) were with hulless caryopsis, of which 869 accessions belonged to six-rowed barley while 47 accessions were of two-rowed barley (Supplementary Figure 1). The awn variability in spike architecture ranged from awnless to very short awned/awnleted elliptical spike to very long awned oblong or fusiform spike in most of the accessions (Figures 2, 3). Awned spikes were predominant in more than 96% of accessions, 2.4% were awnleted, and the remaining were either scurs (Figures 3c,h) or hooded barley (elevated or sessile hoods) (Figures 3l–n). Accession EC492301 was identified for awnless spikes (Figure 3k), which is a rare trait in barley as inflorescences are usually characterized by stiff long bristles/awns. The grain color showed distinct variation for various tonalities of white (6,556), black (134), blue (73), purple (10), and red (5) in both hulled and naked variants, although the white color was predominant in around 97% of accessions (Figure 2 and Supplementary Figure 2).

### Variability for Quantitative Traits and Estimation of the Coefficient of Variation, Heritability, and Genetic Advance

**Figure 4:**
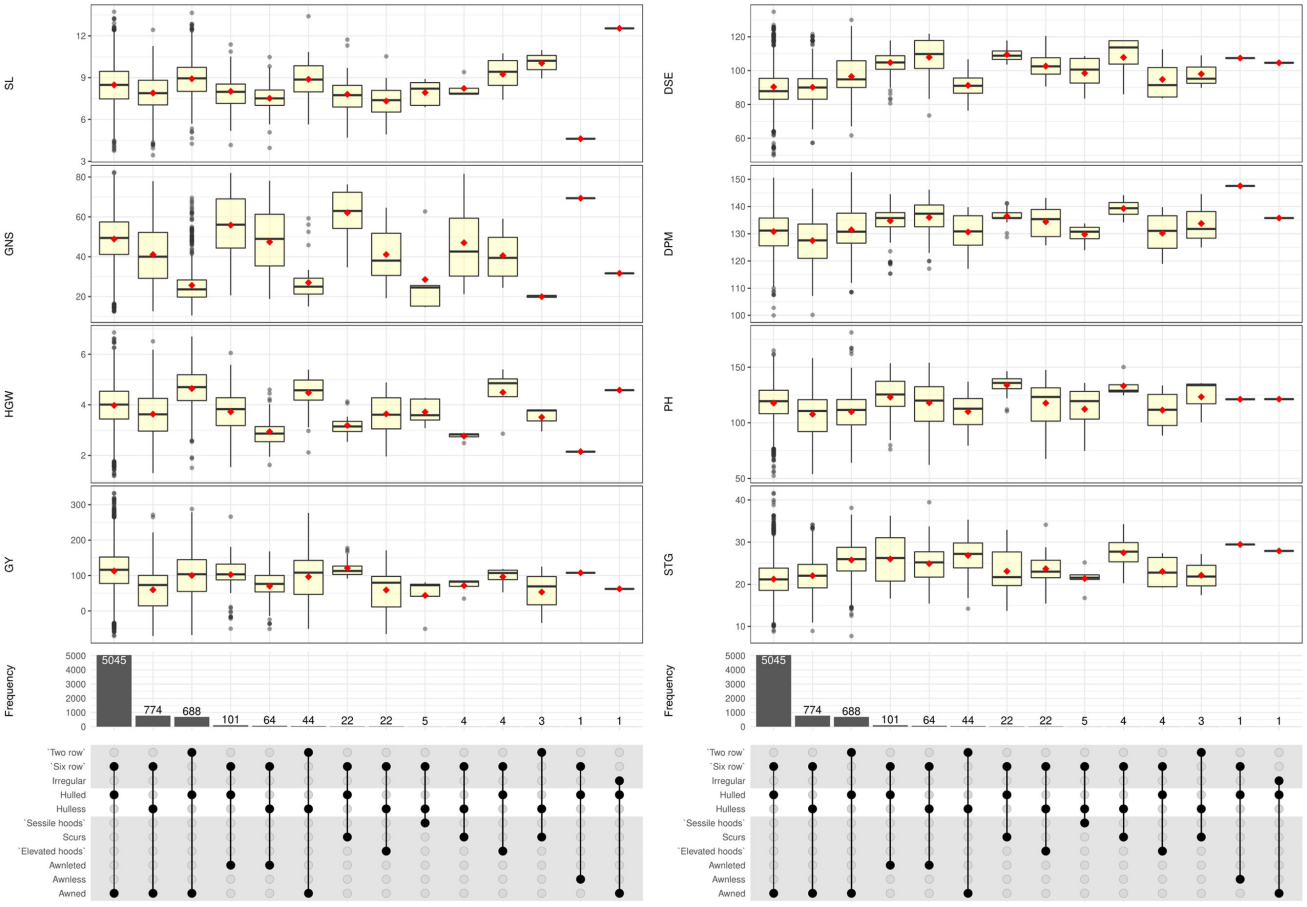
Boxplots showing the distribution of 8 quantitative traits with respect to spike row, grain type, and awn type in 6,778 germplasm accessions of barley.

Wide range of variability was recorded among the accessions as evident from the boxplots presenting distribution of quantitative traits with respect to spike row, grain type, and awn type (Figure 4), and general statistical analysis (Table 1). More than 70% of accessions were intermediate in spike emergence (80–95 days) and maturity (125–140 days). Around 6% accessions were early heading (<75 days) and early maturity (<120 days) while 24% of the total accessions were late heading (>100 days) as evident from frequency distribution plots (Supplementary Figure 3). The mean days to 75% spike emergence ranged from 51 to 139, while the mean DPM ranged from 100 to 152 days. Two-rowed accessions took more days for spike emergence and maturity compared to six-rowed accessions whereas hulless germplasm showed little earliness. The average value of plant height across the entire barley collection was 115.88 cm, however, a range of phenotypes expressing height from 45.96 to 171.32 cm shows good variability for this trait. Around 50 accessions constituting about 0.74% of the total assembly showed dwarf plant habit (<75 cm) which is a highly desirable trait in barley owing to lodging tendency. Mean SL was 8.43 cm, while the range was 3.44–13.73 cm. Mean SL was more in two-rowed accessions (8.95 cm), while it was lesser in hulless germplasm (7.86 cm). Spike triplet groups per spike and the number of grains per spike revealed wide variation ranging from 7.71 to 41.64 triplets and 10.48–82.35 grains/spike, respectively. The most common number of grains per spike was 45–55 grains and occurred in around 50% of barley accessions, which were of six-rowed type. For two-rowed accessions, the number of grains per spike ranged from 25 to 30 in most of the accessions. Large kernel material as indicated by HGW (more than 5.5 g) was recorded in around 2.7% of accessions. HGW showed continuous variation from 1.20 to 6.86 g with an average of 3.99 g. Two-rowed accessions, in general, had higher HGW (4.65 g) followed by six-rowed hulled (3.96 g) and hulless lines (3.58 g). Grain yield was highly variable with an average grain yield per meter row plot of 104.46 g. Hulled accessions out-yielded the hulless ones as indicated by GY of 110.76 g/plot compared to 58.30 g/plot in hulless background. The maximum range of GY was obtained in six-rowed hulled barley (390 g/plot), while it was lesser for two-rowed (282 g/plot) and hulless barley (318 g/plot). The hulless germplasm accessions had in general lesser SL, GNS, HGW, and GY when compared to hulled germplasm.

**Table 1:** General statistical parameters and estimates of genetic diversity among 6,778 barley germplasm accessions.

**Traits**	**Min**.	**Max**.	**Mean ±SE**	**GCV**	**PCV**	**h^**2**^ (bs)**	**GA**	**GAM**
DSE	50.02	134.82	91.46 ± 0.14	11.53	11.88	94.11	21.10	23.07
DPM	99.97	152.57	130.64 ± 0.09	4.60	5.66	65.88	10.05	7.69
PH	45.96	171.32	115.88 ± 0.21	12.89	14.64	77.45	27.11	23.39
SL	3.44	13.73	8.43 ± 0.02	14.49	16.69	75.36	2.19	25.95
STG	7.71	41.64	21.95 ± 0.05	15.61	19.55	63.75	5.64	25.72
GNS	10.48	82.35	45.55 ± 0.18	27.11	30.82	77.40	22.41	49.21
HGW	1.20	6.86	3.99 ± 0.01	20.44	21.54	90.11	1.60	40.04
GY	0^a^	333.17	104.46 ± 0.78	44.92	55.18	66.26	78.80	75.43

*DSE, days to 75% spike emergence; DPM, days to 80% physiological maturity; PH, plant height (cm); NGS, number of grains per spike; SL, spike length (cm); STG, spikelet triplet groups; HGW, hundred-grain weight (g); GY, grain yield per meter row (g); GCV, genotypic coefficient of variation; PCV, phenotypic coefficient of variation, h^2^ (bs), broad-sense heritability; GA, genetic advance; GAM, genetic advance over mean*.

a *Negative adjusted mean values were considered as 0*.

Phenotypic and genotypic coefficients of variation were estimated to know the extent of genetic variation in relation to the environment and genetic factors. PCV and GCV were high for NGS, HGW, GY, and low for DPM, while it was medium for the remaining traits (Table 1). The difference between them is small for all the traits except GY, which is indicative of underlying genetic factors responsible for observed variation and not an environmental role. Heritability was found high for all the traits ranging from 65% for STG to 95% for DSE. The genetic advance was estimated low (<10) for DPM and high (>20) for the rest of the traits. The parameters such as NGS, HGW, GY, SL, PH, and STG showed a high or moderately high level of variance coupled with high heritability and genetic advance.

### Identification of Trait-Specific Germplasm

Evaluation of genetic resources for identification of trait-specific promising donors is important for enhancing the utilization and strategic management of conserved germplasm. Keeping in view, the scarce information available with respect to trait-specific superior accessions in different genetic backgrounds of barley (two-rowed, six-rowed, hulless, hooded *etc*.), the promising accessions were identified and validated (Table 2). These accessions may be used as potential donors for economically important traits to diversify parental material in crop improvement programs.

**Table 2:** Trait specific promising germplasm identified in barley.

**Trait**	**Promising accessions^**$**^**	**Best check (value)**
Days to spike emergence	**IC0138110**, **IC0138116**, EC0578946, EC578711, **IC0113045**, IC0137999^*^, EC0362267^#^, EC0578792^#^ <65 days	BH959 (85) BHS352^#^ (89) DWRB101^*^ (85)
Days to maturity	EC0578370, IC0138109, **IC0138110**, IC0138115, **IC0138116**, IC0138119^*^, IC0138120^*^, IC0138121^*^, **IC0542197**^#^, IC0420720^#^, EC0667490^*^^#^, <115 days	BH959 (128), BHS352^#^ (126) DWRB101^*^ (129)
Plant height (cm)	IC0137895, IC0445980^*^, EC0328964^*^, IC0138111^*^>150 cm **IC0113045**, **IC0138110**, EC0667491^*^^#^ <80 cm	BH959 (120) BHS352^#^ (122) DWRB101^*^ (106)
Spike length (cm)	IC0118656, IC0533203^*^, IC0113052^*^^#^ >12 cm	BH959 (9.08) BHS352^#^ (10.13) DWRB101^*^ (7.96)
Number of grains per spike	IC0438211, EC0578447, EC578458, IC0383651, EC0578521^#^ >75 IC0551180^*^, IC0138171^*^ >30	BH959 (57) BHS352^#^ (56) DWRB101^*^ (26)
Hundred-grain weight (g)	IC0405269, IC0551372, EC0492252^*^, EC0520242^*^, IC0138120^*^, IC0138111^*^, **IC0542197**^#^ >5.5 g	BH959 (4.49) BHS352^#^ (3.22) DWRB101^*^ (5.03)

$ *Accessions in bold represent superiority for more than one trait*;

* *Two-rowed accessions*;

# *Hulless accessions*.

### Quality Evaluation of the Core Collections Developed Using MSTRAT, PowerCore, and Core Hunter 3

The comparison of evaluation indices for checking the quality of seven core sets developed are presented in Table 3. The core sets developed by MSTRAT and PowerCore showed the least value of genetic distances E-NE, E-E, average Shannon-Weaver diversity index (H'), and other indices based on mean and variance such as MD% and VD%, which is indicative of capturing less diversity.

**Table 3:** Comparison of different core sets developed based on core quality evaluation indices.

**Criteria**	**MSTRAT**	**PowerCore**	**Core Hunter 3**				
			**Core set-1**	**Core set-2**	**Core set-3**	**Core set-4**	**Core set-5**
E-NE	0.053	0.059	0.099	0.060	**0.085**	0.091	0.078
A-NE	0.130	0.133	0.138	0.122	**0.126**	0.129	0.124
E-E	0.216	0.251	0.277	0.237	**0.267**	0.271	0.259
MD%	25	62.5	87.5	62.5	**62.5**	75	87.5
VD%	12.5	100	100	50	**87.5**	62.5	87.5
CR%	85.72	99.61	97.93	88.26	**94.27**	90.47	96.33
VR%	104.1	122.9	121.8	100.5	**113.8**	109.6	117.7
Average H'	0.568	0.651	0.749	0.658	**0.735**	0.719	0.729
Class Coverage	90	90	90	90	**90**	90	90
Mantel correlation	0.938^**^	0.949^**^	0.928^**^	0.958^**^	0.949^**^	0.953^**^	0.949^**^

*E-EN, the average distance between each entry and nearest neighboring entry; A-NE, the average distance between each accession and the nearest entry; E-E, the average genetic distance between entries; MD%, Mean difference percentage; VD%, variance difference percentage; CR%, coincidence rate of range; VR%, variable rate of range; H', Shannon diversity index*,

** *indicates significance at p = 0.01*.

The optimum value of all three genetic distances i.e., maximized E-NE and E-E along with minimization of A-NE is shown by core set-3 developed by using CH3. Furthermore, the CH3-core set 3 had VD 87.5%, CR 94.27%, and VR 113.8%), which were more than the threshold value of VD (80%), CR (80%), and VR (100%) and high value of average Shannon-Weaver diversity index (H') required for a good core collection. Although the MD in CH3-core set 3 was 62.5% which is more than the desired value. The inter-relationships between traits were conserved in all the analyzed core sets with reference to the whole collection as indicated by Mantel correlation. Based on various evaluation indices described above, the CH3-Core set-3 (hereafter referred to as INGB barley core set) appeared to capture the prevalent diversity and representativeness of the whole collection to a maximum extent. Therefore, we selected this core set for further comparative analysis with the entire collection. This core set was constituted by 678 accessions comprising 521 accessions of six-rowed and 157 two-rowed barley including 220 accessions having hulless caryopsis. List of INGB core set accessions along with the source of collection sites, biological status, and characterization data on 9 qualitative and 8 quantitative traits is presented in Supplementary Table 2. The INGB core set accessions can be shared across the globe as per extant national legislation and international agreement.

### Comparative Evaluation of the INGB Barley Core Set With the Whole Collection

**Figure 5:**
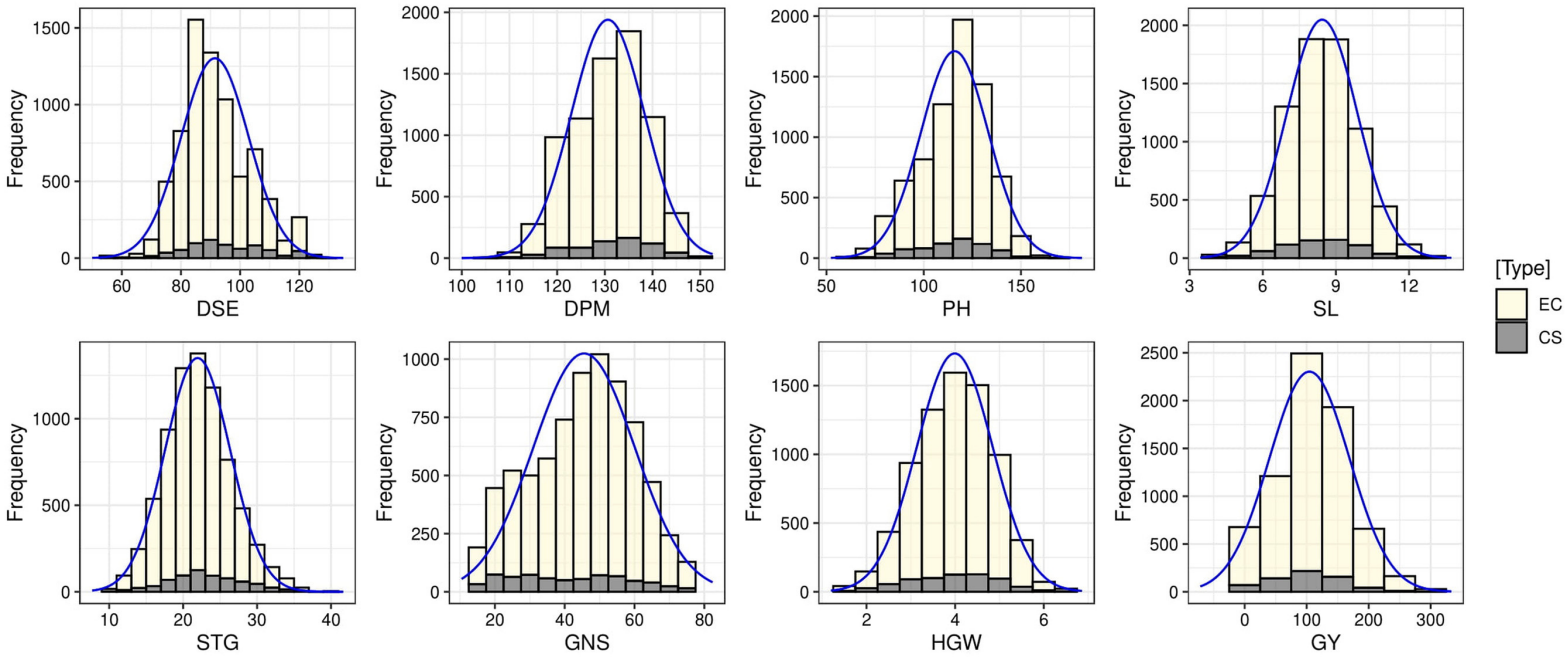
Frequency distribution plots showing the comparison of variability of quantitative traits in the entire collection (EC) and core set (CS) of barley.

The means, ranges, CV, interquartile range, and frequency distribution of different quantitative traits among the selected core set and entire collection are given in Table 4 and box plots in Figure 4. We could obtain a higher CV for all the traits in the core set as compared to the whole collection which is indicative of capture of more variability in the core set. The difference in the means of the core set and the whole collection was non-significant for SL and HGW as revealed by the Newman-Keuls test, however, the *t*-test provided a non-significant difference for three traits (DPM, SL, and HGW). Levene's test revealed non-significant differences among variances for traits PH and GY, and significant for other traits such as DSE, DPM, SL, STG, GNS, and HGW. Frequency distribution plots (Figure 5) and boxplots (Supplementary Figure 4) depicting the quantum of quantitative trait variability captured showed the representation of all classes from the entire collection in the core set. The interquartile range was almost similar for all the traits such as DPM, HGW, PH, SL, STG, and GY, except DSE and NGS (Table 4) which show a symmetrical distribution of accessions for the majority of the traits among the core set and the entire collection. We also generated QQ plots and computed Kullback-Leibler distance to see the distribution pattern of all the 8 qualitative traits in both the core set and the whole collection (Supplementary Figure 5). The range of Kullback distances from 0.024 to 0.071 for all traits indicated the distribution of traits such as DPM, PH, SL, STG, HGW, and GY in the core collection was identical to the whole collection.

**Table 4:** Comparison of range, mean, coefficient of variation, interquartile range, and frequency distribution in the entire collection and core set for various quantitative descriptors used in the formation of core collection in barley.

**Traits**	**Entire collection**					**Core set**					**Differences between core set and entire collection**			
	**Min**	**Max**	**Mean ±SE**	**Coeff. of variation**	**Inter-quartile range**	**Min**	**Max**	**Mean ±SE**	**Coeff. of variation**	**Inter-quartile range**	**Mean^**a**^**	**Mean^**b**^**	**Variance^**c**^**	**Frequency distribution^**d**^**
DSE	50.02	134.82	91.46 ± 0.14	11.88	14.6	50.02	130.02	94.73 ± 0.51	13.90	19.2	^**^	^**^	^**^	^**^
DPM	99.97	152.57	130.64 ± 0.09	5.66	11.0	100.17	152.57	131.28 ± 0.32	6.42	12.15	^*^	ns	^**^	^**^
PH	45.96	171.32	115.88 ± 0.21	14.64	23.21	62.24	167.40	113.88 ± 0.69	15.83	25.7	^**^	^**^	ns	^**^
SL	3.44	13.73	8.43 ± 0.02	16.69	1.96	3.44	13.41	8.33 ± 0.06	19.20	2.21	ns	ns	^**^	ns
STG	7.71	41.64	21.95 ± 0.05	19.55	5.67	7.71	39.44	23.06 ± 0.19	21.90	6.8	^**^	^**^	^**^	^**^
GNS	10.48	82.35	45.55 ± 0.18	30.82	20.67	12.48	81.61	41.64 ± 0.64	40.02	27.06	^**^	^**^	^**^	^**^
HGW	1.20	6.86	3.99 ± 0.01	21.54	1.20	1.45	6.70	3.93 ± 0.04	24.71	1.43	ns	ns	^**^	ns
GY	0^a^	333.17	104.46 ± 0.78	55.18	78.96	0^a^	319.84	91.17 ± 2.53	72.16	81.59	^**^	^**^	ns	^**^

*DSE, days to 75% spike emergence; DPM, days to 80% physiological maturity; PH, plant height (cm); NGS, number of grains per spike; SL, spike length (cm); STG, spikelet triplet groups; HGW, hundred-grain weight (g); GY, grain yield per meter row (g); ^a^Negative adjusted mean values were considered as 0*.

a *Differences between means of entire collection and core set were tested by Newman–Keuls test*.

b *Differences between means of entire collection and core set were tested by t-test*.

c *Variance homogeneity as tested by Levene's test*.

d *Difference of frequency distribution by Wicoxon rank test*.

*ns indicate not significant*;

* and ** *indicate significant differences at 5% and 1% probability level, respectively*.

For qualitative or categorical data, we calculated the Shannon-Weaver diversity index (H') in the core set and entire collection (Table 5). The extracted core set confirmed maximization of existing diversity as shown by the increased value of H' for all the traits except for a very small difference observed for SD and LT. Both of these traits (SD and LT) are already expressing the maximum diversity in both the entire collection (1.070 and 1.361) and core set (1.068 and 1.318), respectively, which is quite close to the maximum possible value (H' max) of SD (1.099) and LT (1.386). An increase in the H' based evenness values across all the traits in the core is indicative of effective representativeness of diversity available in the whole collection of barley.

**Table 5:** Shannon diversity index of qualitative traits in the entire collection and core set of barley germplasm.

**Descriptor**	**Shannon-Weaver diversity index (H')**		**H' max**		**Evenness**	
	**Entire collection**	**Core set**	**Entire collection**	**Core set**	**Entire collection**	**Core set**
GC	0.204	0.439	1.099	1.099	0.186	0.400
EPV	0.297	0.608	1.099	1.099	0.271	0.554
GH	0.651	0.832	1.099	1.099	0.593	0.758
SR	0.345	0.550	1.099	1.099	0.314	0.501
AT	0.175	0.536	1.792	1.792	0.097	0.299
SD	1.070	1.068	1.099	1.099	0.974	0.972
LT	1.361	1.318	1.386	1.386	0.982	0.951
GT	0.415	0.720	1.099	1.099	0.378	0.655
GPC	0.174	0.544	1.609	1.609	0.108	0.338

*GC, growth class; EPV, early plant vigor; GH, growth habit; SR, spike row type; AT, awn type; SD, spike density; LT, lodging tendency; GT, grain type; GPC, grain pericarp color*.

### Correlation and Principal Component Analysis (PCA)

The estimates of trait associations revealed that significant and positive correlations exist among the traits such as DSE and DPM (*r* = 0.58 in the entire collection, *r* = 0.69 in the core set) and between PH and GY (*r* = 0.53 in entire collection, *r* = 0.52 in core set) (Supplementary Table 3). PH was also significantly and positively correlated with other yield components such as SL, GNS, and STG in both the entire collection as well as the core set. This indicates that PH is directly associated with the yield potential of the plant in addition to being an important fodder trait in barley. Among all 28 pairwise comparisons (*r*-values) possible with eight quantitative traits, 26 were significant in the entire collection and 23 in the core set, and were conserved after sampling of the core set in terms of trait association as well as magnitude.

Principal component analysis (PCA) based on the correlation between eight quantitative traits was done to examine the spatial distribution of entries/accessions in the core set and entire collection. The first five principal components (PCs) explained the major portion of variance (78%) in the barley core set. This value was comparable with that of the entire collection (76%) (Table 6). In addition, the proportion of variance explained by individual PCs was quite comparable in the core set and whole collection. Factor loadings for the traits under consideration showed that PH, SL, STG, GY, and NGS contributed mostly to PC1; DSE and DPM contributed to PC2; GW and HGW contributed to PC3 for both entire and core collection in barley germplasm.

**Table 6:** Comparison of first five principal components in entire collection and core set of barley germplasm.

**Principal components**	**Entire collection**			**Core set**		
	**Standard deviation**	**Proportion of variance**	**Cumulative proportion**	**Standard deviation**	**Proportion of variance**	**Cumulative proportion**
PC1	1.55	0.24	0.24	1.55	0.24	0.24
PC2	1.28	0.16	0.41	1.35	0.18	0.42
PC3	1.18	0.14	0.55	1.18	0.14	0.56
PC4	1.07	0.12	0.66	1.07	0.11	0.68
PC5	0.99	0.10	0.76	1.00	0.10	0.78

## Discussion

### Phenotypic Diversity of the Crop and Identification of Trait-Specific Desirable Accessions

Germplasm is the basic need for any crop improvement program as it is the base of genetic variability enriched with valuable genes. Several researchers have emphasized the characterization of genetic resources conserved into genebanks is the pivotal step to gain insight into the traits possessed by germplasm collections. Our study helped us in categorizing the barley collection under study into different categories such as two-rowed for malt, six-rowed for feed, hooded for fodder, hulless for food *etc*., which forms the basic platform for detailed evaluation for feed, food, and malt purpose and also colored barley for functional food purpose *per se*. The high variability recorded in spike and grain traits in the present study shows the remarkable adaptability in terms of morphological structure and environmental conditions in which barley is cultivated throughout the world (Sarkar et al., 2010; Dawson et al., 2015; Verma, 2018). Earlier reports by Manjunatha et al. (2007) and Sarkar et al. (2010) show congruence for the predominance of qualitative traits reported here, however rare or less common phenotypic traits such as awnless spike, hulless caryopsis variant in two-rowed spike, hooded inflorescence as well as colored caryopsis in hulled and hulless background were not reported in previous studies. Mainly awned barley varieties are grown for production purposes (Tsvetkov and Tsvetkov, 2007) and awnless forms (*lks1*) are largely unknown (Yuo et al., 2012). Under agricultural conditions, the long and stiff awn is not a desirable trait because they hinder manual harvesting, storage, even reduce the feed value of straw for livestock by being potentially injurious (Takahashi, 1955; Yuo et al., 2012). Thus, the awnless trait (Figure 3k) can be exploited in selection efforts to reduce awn length and to unravel the mechanism of awn elongation in grasses. Hooded inflorescence represents another variation in the spike structure in which awns are transformed into an extra flower of inverse polarity. The hooded inflorescence is desirable for fodder purpose barley as hoods allow more palatability and lip-smacking in animals. We recorded a total of 31 hooded accessions among which 5 accessions showed sessile hoods and 26 accessions had elevated hoods (Figure 2), most of which were hulless (27 accessions) (Figure 4). Moreover, the hooded accessions had high PH (>100 cm) and biomass with more leaf length and leaf width. The information on hulless grain traits in two-rowed barley is scarce and scattered. We characterized 47 accessions as having hulless caryopsis in two-rowed background. These accessions had desirable traits such as more SL, STG, HGW, GY, bold grains, and less lodging tendency compared to six-rowed hulless barley (Figure 4). Likewise, scarce information is available globally on the occurrence and evaluation of colored barley. We recorded distinct variation in grain pericarp color (Figure 2) in affinity with the findings reported by Hua et al. (2015) in National Barley Improvement Centre of China. The colored caryopses being rich in anthocyanins and phenolic compounds are recognized as promising ingredients in cereal-based functional food and natural coloring agents (Abdel-Aal et al., 2006; Kim M. J. et al., 2007), for extracting the alcoholic beverage “chang” with its distinct flavor and color (Tashi et al., 2012), and associated lower *Fusarium* head blight incidence (Choo et al., 2005). Besides, darkly colored barley is also preferred by certain farming communities, for example, Syrian farmers believe that black barley is more palatable to sheep compared to white as feed, while Tibetan farmers prefer to grow black barley due to its adaptive background in drought-prone environments.

We recorded good variability and range of phenotypic expression for quantitative traits. Our study identified around 6.04% accessions as early heading (<75 days) of which 1% (65 accessions) showed extra early heading. For maturity also, around 6.26% of accessions showed early maturity in less than 120 days, among which only 1.08% (73 accessions) attained maturity in <115 days. Early types are essential in most barley-producing areas owing to rain-fed cultivation. In India, about 44% of the area under barley cultivation is rain-fed. Hence early maturity is an obvious breeding target to escape drought and terminal heat stress at the end of the growing season. Besides this, the short duration and fast-growing genotypes of barley perfectly fit the major cropping system cereal-legume (for food and feed) in dryland areas of arid/semi-arid zones. About 24% of the total accessions were late heading (>100 days) of which 40% were exotic, mainly received from Syria and Mexico. This may be due to the fact that winter in these countries is much prolonged and cooler and selection is for late plant types to escape frost damage. Around 50 accessions constituting about 0.74% of the total assembly showed dwarf plant height (<75 cm), which is a highly desirable trait as barley is a lodging-prone crop. Although studies on diversity assessment in Indian barley (Manjunatha et al., 2007; Sarkar et al., 2010; Kumar et al., 2015; Grewal and Kaur, 2018; Kaur et al., 2018; Manju et al., 2019; Yadav et al., 2020) have shown various levels of phenotypic expression and variability, nonetheless, the data generated are often limited to few germplasm accessions or traits and thus gave an incomplete overview of the available variability. A similar range of phenotypic expression for major agro-morphological traits in barley was reported by Manjunatha et al. (2007) and Sarkar et al. (2010) in Indian germplasm. In exotic barley germplasm a varying degree of agro-morphological and phenological variability, albeit with a lower magnitude was also reported by several authors (Lasa et al., 2001; Assefa and Labuschagne, 2004; Abebe et al., 2008, 2010; Ahmad et al., 2008), which may be due to lesser number of germplasm accessions studied.

High GCV coupled with high heritability and genetic advance is considered useful for prediction based on phenotypic performance (Johnson et al., 1955). The parameters such as GNS, HGW, GY, SL, PH, and STG showed high or moderately high levels of variance coupled with high heritability and genetic advance (Table 1). This suggests that these are inherited traits and most likely the heritability is due to additive gene effects and selection may be more effective. Therefore, these characteristics may be subjected to different selection schemes for exploiting additive gene action to produce widely adapted genotypes. On the other hand, days to maturity has low estimates of variability and genetic advance which means that the effect of environmental conditions is more on these traits as compared to other traits. These are under the influence of non-additive gene interaction and need to be improved by hybridization. High value of these traits was shown by six-rowed accessions, namely EC0578515, EC0578829, IC0138114, IC0057532, IC0113045, EC0492282, IC0283590, IC0118656; two-rowed accessions IC0138120, IC0138121, EC0177231, IC0533203, IC0533206, IC0533203, EC0328942; and hulless lines EC0578609, IC0082589, IC0082593, EC0667492, IC0113052, IC0073636, IC0041585, IC0445822, which suggests that these accessions can be subjected to yield gain in barley through simple phenotypic selection. Our observations are in consonance with earlier results by Verma et al. (2007); Eshghi et al. (2011), and Marzougui and Chargui (2018), who reported high heritability for the grain number/spike, grain yield, thousand kernel weight, plant height, and spike length, however Baghizadeh et al. (2003) and Rohman et al. (2006) observed non-additive type of inheritance for GNS in barley.

### INGB Barley Core Collection Effectively Captures Maximum Diversity and Representativeness of the Whole Collection

The enormous increase in the number and size of *ex-situ* germplasm collections over the past few decades have posed complications for the evaluation, utilization, and maintenance of genetic resources. Therefore, the concept of forming the core collections was introduced to increase the efficiency of evaluation and utilization of stored collections, while preserving the maximum genetic diversity of the whole collection (Frankel, 1984; Brown, 1989). Two general purposes of the creation of core collections (i) maximizing the total genetic diversity in a core (favored by taxonomists, geneticists, and genebank curators), and (ii) maximizing the representativeness of the genetic diversity (preferred by plant breeders) have been suggested by Marita et al. (2000). However, users have a strong preference for optimizing the chances of finding new accessions/alleles in an adapted background as sought in breeding programs instead of only maximizing diversity *per se*, hence there comes the aspect of balance between representing the total diversity and usefulness of the core. Keeping researcher's desire to have a single multipurpose core set with maximum diversity and representativeness in view, we used different approaches–MSTRAT (Gouesnard et al., 2001), PowerCore (Kim K. W. et al., 2007), and Core Hunter (Thachuk et al., 2009; De Beukelaer and Davenport, 2018; De Beukelaer et al., 2018) for the development of core set and evaluated the resultant seven independent core sets on basis of various criteria (Odong et al., 2013). The detailed comparative statistical analyses (Table 3) suggested that the CH3-Core set three developed by maximizing both E-NE and A-NE with equal weightage of 1:1 shows maximum diversity as shown by high E-NE and E-E along with maximum representativeness as evident from low A-NE genetic distances. Maximizing the average genetic distance between entries of a core collection has been suggested as a desired quality criterion for core collections intended for plant breeders (Franco et al., 2006; Thachuk et al., 2009). Further, the assessment of the difference between means, variance, and range of the whole collection and different core sets represented by MD%, VD%, CR%, and VR%, indicated that INGB core had 87.5% VD, 94.27% CR, and 113.8% VR. For a core collection to be more diverse and representative, a lower value of MD (<20%), larger VD and CR (>80%), and VR (>100%) is desirable (Hu et al., 2000; Agrama et al., 2009). Evaluation of core sets using similar parameters was also reported by Nanjundan et al. (2021) in Indian mustard, Phogat et al., 2021 in wheat, Archak et al. (2016) in chickpea, Kumar et al. (2016) in Safflower and (Agrama et al., 2009) in rice core sets. Geographical representativeness of extracted core set is evident from the relative distribution of sources/areas of collection of indigenous and exotic germplasm in entire collection and core set presented in Supplementary Figure 6.

For detailed quality evaluation, the selected core set was further compared with the whole collection of barley germplasm based on summary statistics (comparison of the mean, variance, CV, interquartile range, frequency distribution), Shannon-Weaver diversity index, correlation coefficient analysis, and PCA. We could obtain a higher CV for all the traits in the core set as compared to the whole collection which is indicative of capture of more variability in the core set. The interquartile range was almost similar for all the traits such as DPM, HGW, PH, SL, STG, and GY, except DSE and GNS (Table 4) which shows the symmetrical distribution of accessions for a majority of the traits among the core set and entire collection. The relative frequency bar plots for qualitative traits in the whole collection and core set show the capture of the whole range of variation (Supplementary Figure 7). Similar inference could be drawn from the boxplots showing the distribution of eight quantitative traits in the entire collection and core set of barley (Supplementary Figure 4). The use of quantile-quantile (QQ) plots (Wilk and Gnanadesikan, 1968) and probability distribution-based methods such as Kullback-Leibler distance (Kullback and Leibler, 1951) has been designated as one of the best options for the evaluation of the core set for maximum representation of the pattern of variation in the entire collection. In our case, from both the QQ plots and Kullback-Leibler distance (ranging between 0.024 and 0.071 for all traits) it is evident that the distribution of traits such as DPM, PH, SL, STG, HGW, and GY in the core collection was identical to the whole collection (Supplementary Figure 5) as a minimum is the value of Kullback distance, more identical is the distribution of a trait in the core set with reference to the whole set. The extracted core set also confirmed maximization of existing diversity as shown by the increased value of H' for all the traits except very small difference observed for SD and LT, both of which are already expressing the maximum possible value (H' max) in the entire collection and core set (Table 5).

Correlation coefficient analysis had been used widely in many crop species including barley to determine inter-relationships among different traits under study, particularly the traits that directly affect the grain yield (Babaiy et al., 2011; Al-Tabbal and Al-Fraihat, 2012; Kaur et al., 2018). The correlation coefficients showed a strong positive correlation among the traits such as DSE and DPM, PH and GY, SL and STG, DPM, and PH in the whole collection as well as the core set (Supplementary Table 3). The comparison of r values (Supplementary Table 3) showed that association between traits has been conserved well enough in the core collection. The comparison between correlation coefficients of the whole collection and the core collection has been used as a criterion for evaluating the quality of core collections (Reddy et al., 2005; Mahajan et al., 2007; Archak et al., 2016). The significant positive correlation between PH and GY (*r* = 0.53, P_0.05_) indicated that plant height is directly associated with the yield potential of the plant in addition to being an important fodder trait in barley. In addition, PH was also significantly and positively correlated with other yield components such as SL, GNS, STG. These results are in congruence with earlier reports by Jabbari et al. (2010), Birol and Necmettin (2011), Drikvand et al. (2011), Ruzdik et al. (2015), and Kaur et al. (2018). Grain number per spike and thousand seed weight are important yield components that are usually used as a selection trait in barley breeding. In our germplasm, these yield-attributing components such as GNS, HGW, SL, and STG were positively correlated with each other as well as with GY as also reported by Akdeniz et al. (2004); Jabbari et al. (2010); Drikvand et al. (2011), and Ruzdik et al. (2015), and suggesting that more emphasis should be given to these traits while performing selection intended to improve grain yield in barley.

PCA offers another exploratory criterion for evaluating core collections by the inspection of the spatial distribution of the entries in plots of principal components (Bisht et al., 1998; Kang et al., 2006; Mahajan et al., 2007). Core collection can be related to the whole collection based on the sum of squares of the scores of the entries on the major principal components, where a greater value indicates a more diverse core collection (Noirot et al., 1996). In the present study, PCA helped us to assess the relatively high contribution of traits such as plant height, grains/spike, spike length, grain yield, days to spike emergence, and maturity loaded on major PCs representing total variance, which is in agreement with Manjunatha et al. (2007); Ebrahim et al. (2015), and Kaur et al. (2018) who also reported high contribution of these traits while assessing genetic diversity in barley. The proportion of variance explained by individual PCs was quite comparable and conserved in the core set and whole collection as evident from Table 6.

Thus, it is evident from the results explained here that a wide spectrum of variability for agro-morphological and phenological traits does exist in barley germplasm assembly at INGB, the magnitude of which is conserved in the extracted core set. The results reinforce the agro-morphological characterization-based assessment of genetic variability as a pivotal starting point for the efficient utilization and conservation of germplasm holdings amid reduced total genetic diversity.

## Conclusions

This report summarizes the results on the characterization of 6,778 barley accessions, including 1,427 exotic introductions conserved in the National Genebank of India based on agro-morphological and phenological traits. Statistical analysis of distribution parameters showed a wide range of phenotypic expression for the traits such as DSE, PH, SL, GNS, HGW, and GY in the barley germplasm. The identification of promising accessions for agronomically important traits such as earliness, plant height, spike length, hundred-grain weight, and grain yield in different genetic backgrounds (six-rowed, two-rowed, hulless) may be utilized in future selection and breeding in barley to cater to different agro-ecologies and end-uses. Based on the genetic variability laid out in the present study, the INGB barley core set comprising 678 accessions has been developed after comparative quality evaluation of seven different core sets derived using different software and strategies. The core set captured maximum diversity and representativeness of the whole collection as evident from various evaluation indices based on comparison of mean, variance, range (MD%, VD%, CR%, VR%, interquartile range), QQ plots, Shannon-Weaver diversity index along with the optimisation of average genetic distance described in this report. The development of barley core set and trait-specific germplasm identified in this study will provide expedited access to genetically diverse and agronomically important genetic resources, which would be useful in widening the genetic base of barley and thus more effective breeding programs. Besides the diverse germplasm in the form of the core set may be subjected to field evaluation for various biotic and abiotic stresses and subsequently genome-wide association studies to identify the underlying genes/alleles/markers.

## Data Availability Statement

The original contributions presented in the study are included in the article/Supplementary Material, further inquiries can be directed to the corresponding author.

## Author Contributions

VK: conceived, designed and conducted the experiment, collected and compiled the data, and wrote the manuscript. JA: analyzed the data. M and JK: sowing and collection of phenotypic data. SJ: provided seed material from National Genebank. BP: facilitation for completion of the experiment. NP: layout, sowing, and collection of phenotypic data. JR: reviewed the manuscript. AP: conservation of diverse specimen in National Herbarium of Cultivated Plants and NBPGR and reviewed the manuscript. AK: facilitation and guidance for the success of large-scale characterization of germplasm. All authors contributed to the article and approved the submitted version.

## Funding

This study was supported by institutional funding resources from ICAR-NBPGR Institutional Project (PGR/GEV-BUR-DEL-01.01).

## Conflict of Interest

The authors declare that the research was conducted in the absence of any commercial or financial relationships that could be construed as a potential conflict of interest.

## Publisher's Note

All claims expressed in this article are solely those of the authors and do not necessarily represent those of their affiliated organizations, or those of the publisher, the editors and the reviewers. Any product that may be evaluated in this article, or claim that may be made by its manufacturer, is not guaranteed or endorsed by the publisher.

## Supplementary Material

Supplementary Figure 1 Distribution of 6,778 germplasm accessions of barley in different categories with respect to spike row, grain type, and awn type.

Supplementary Figure 2 Variability in grain shape, size, and colour in barley germplasm.

Supplementary Figure 3 Frequency distribution plots of quantitative traits in barley.

Supplementary Figure 4 Boxplots showing the distribution of 8 quantitative traits in the entire collection (EC) and core set (CS) of barley.

Supplementary Figure 5 Quantile-Quantile (QQ) plots and Kullback-Leibler distance for the entire collection and core set for quantitative traits.

Supplementary Figure 6 Relative geographic representation of collection sites of barley germplasm in the entire collection (EC) and core set (CS).

Supplementary Figure 7 Relative frequency bar plots of qualitative traits in the entire collection (EC) and core set (CS).
