# Supplementary figures and images for: Phenotypic Characterization, Genetic Diversity Assessment in 6,778 Accessions of Barley (Hordeum vulgare L. ssp. vulgare) Germplasm Conserved in National Genebank of India and Development of a Core Set
Source: Front Plant Sci. 2022 Feb 24;13:771920. doi: 10.3389/fpls.2022.771920 (PMC8913045; doi:10.3389/fpls.2022.771920)

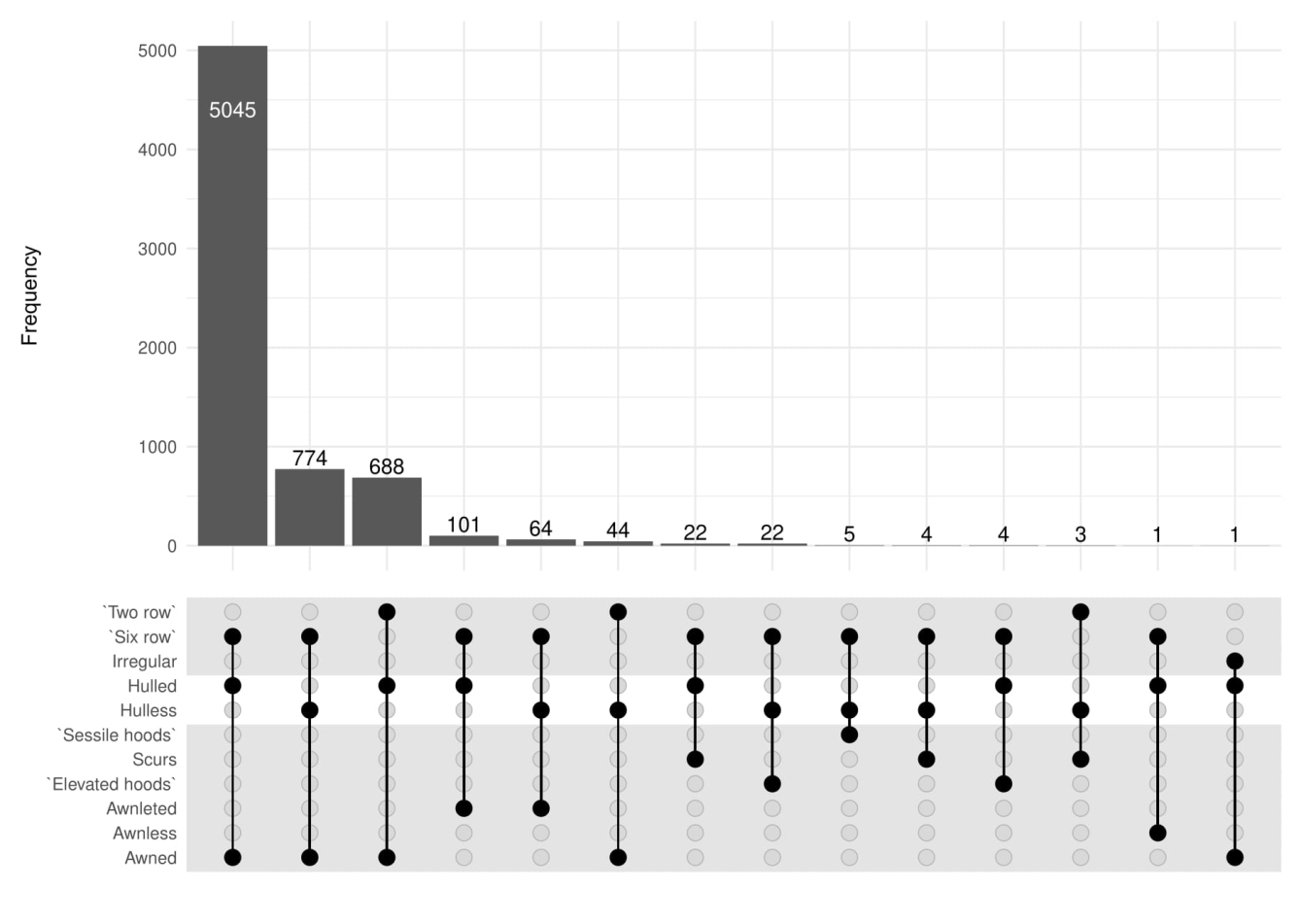

Supplement: Supplementary Figure 1 — Distribution of 6,778 germplasm accessions of barley in different categories with respect to spike row, grain type, and awn type. [file Image_1.TIF]

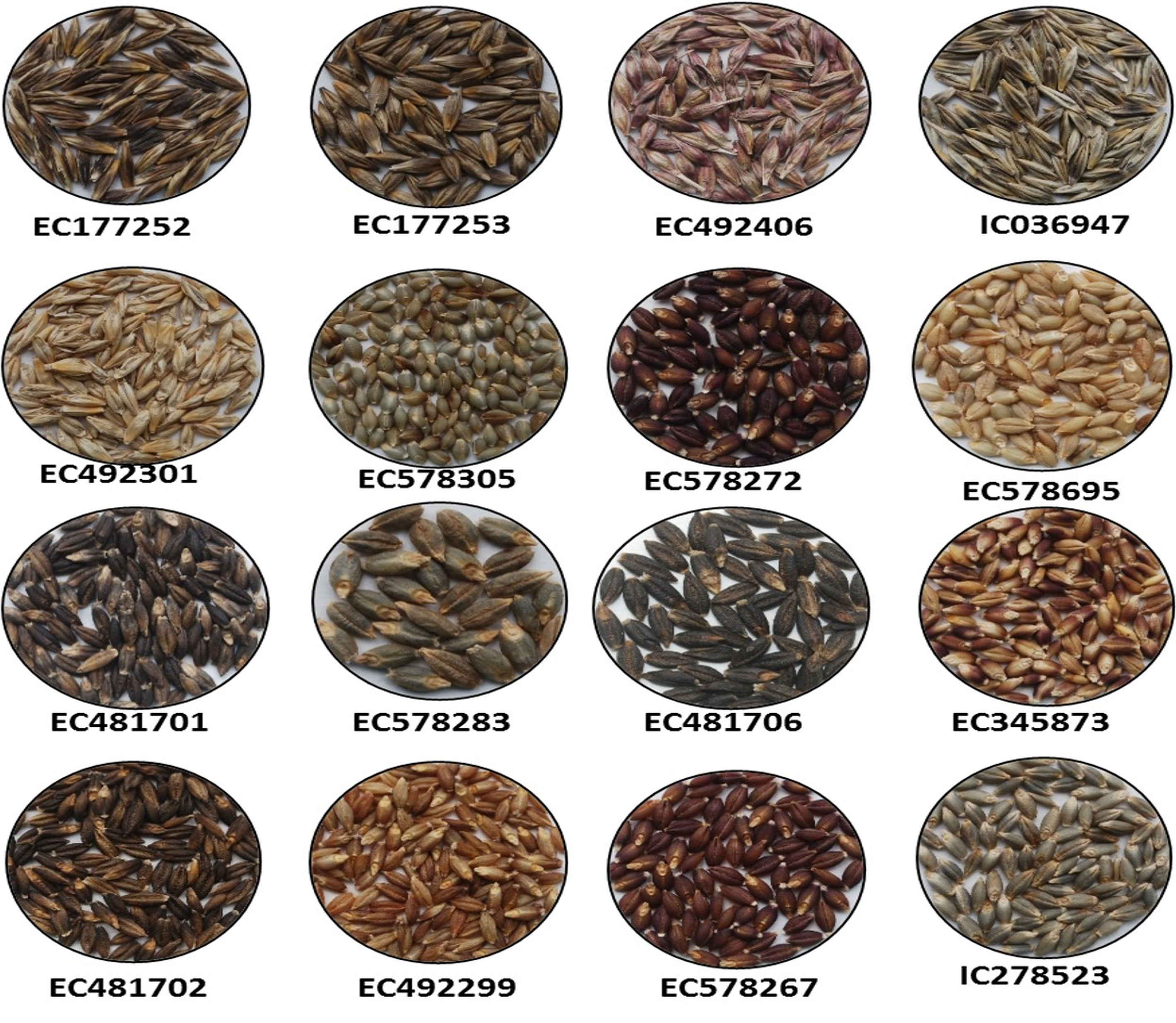

Supplement: Supplementary Figure 2 — Variability in grain shape, size, and colour in barley germplasm. [file Image_2.TIF]

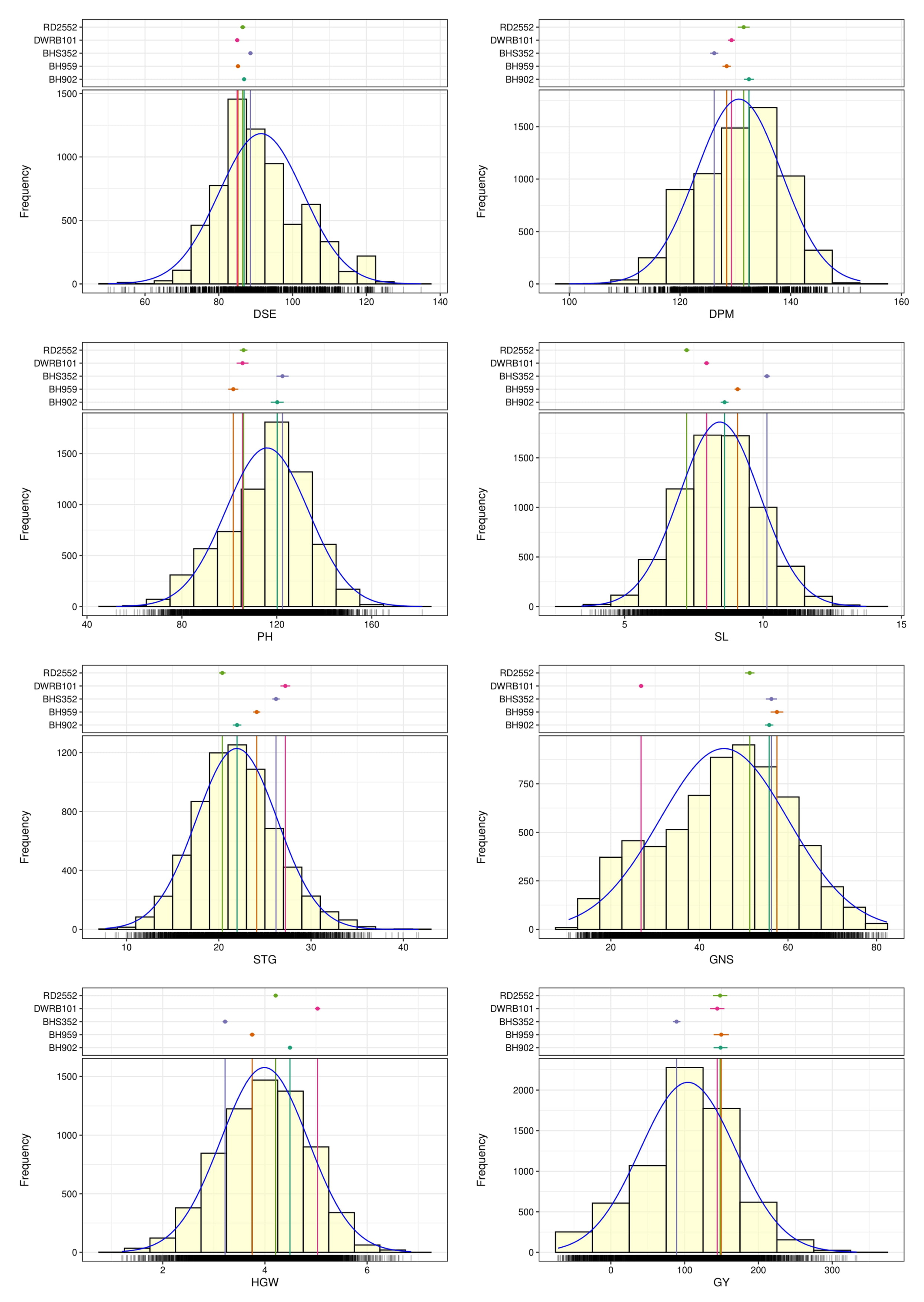

Supplement: Supplementary Figure 3 — Frequency distribution plots of quantitative traits in barley. [file Image_3.TIF]

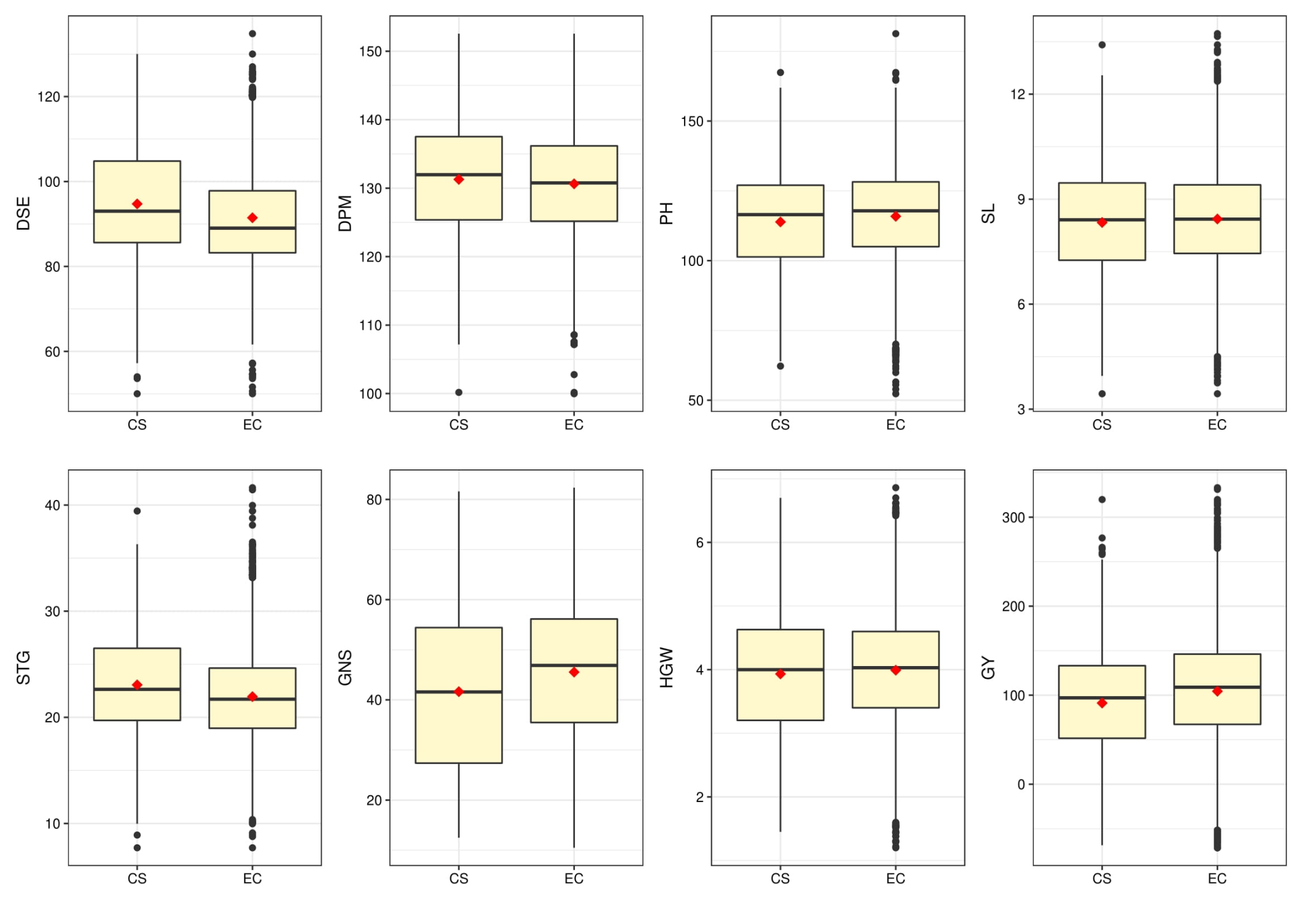

Supplement: Supplementary Figure 4 — Boxplots showing the distribution of 8 quantitative traits in the entire collection (EC) and core set (CS) of barley. [file Image_4.TIF]

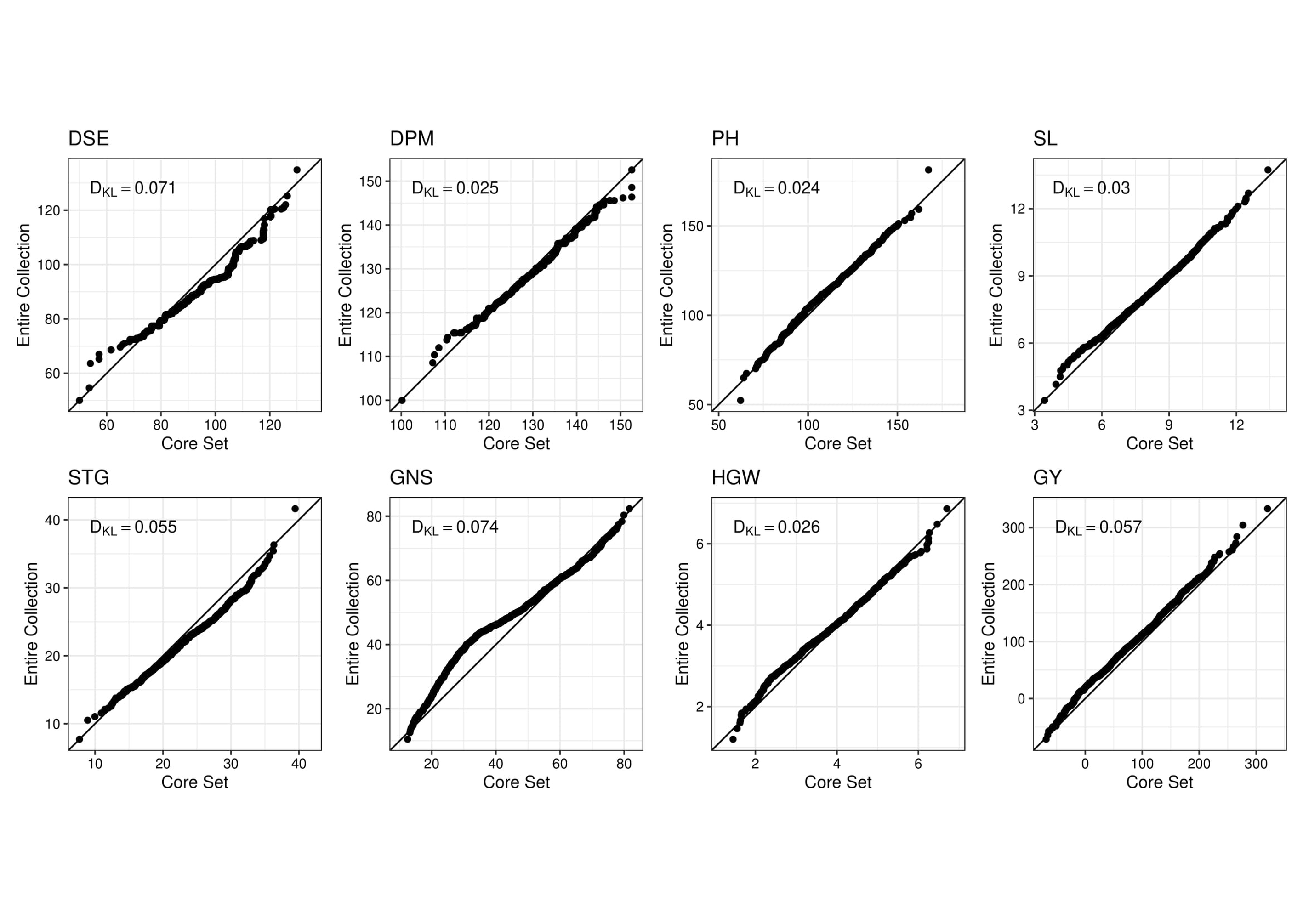

Supplement: Supplementary Figure 5 — Quantile-Quantile (QQ) plots and Kullback-Leibler distance for the entire collection and core set for quantitative traits. [file Image_5.TIF]

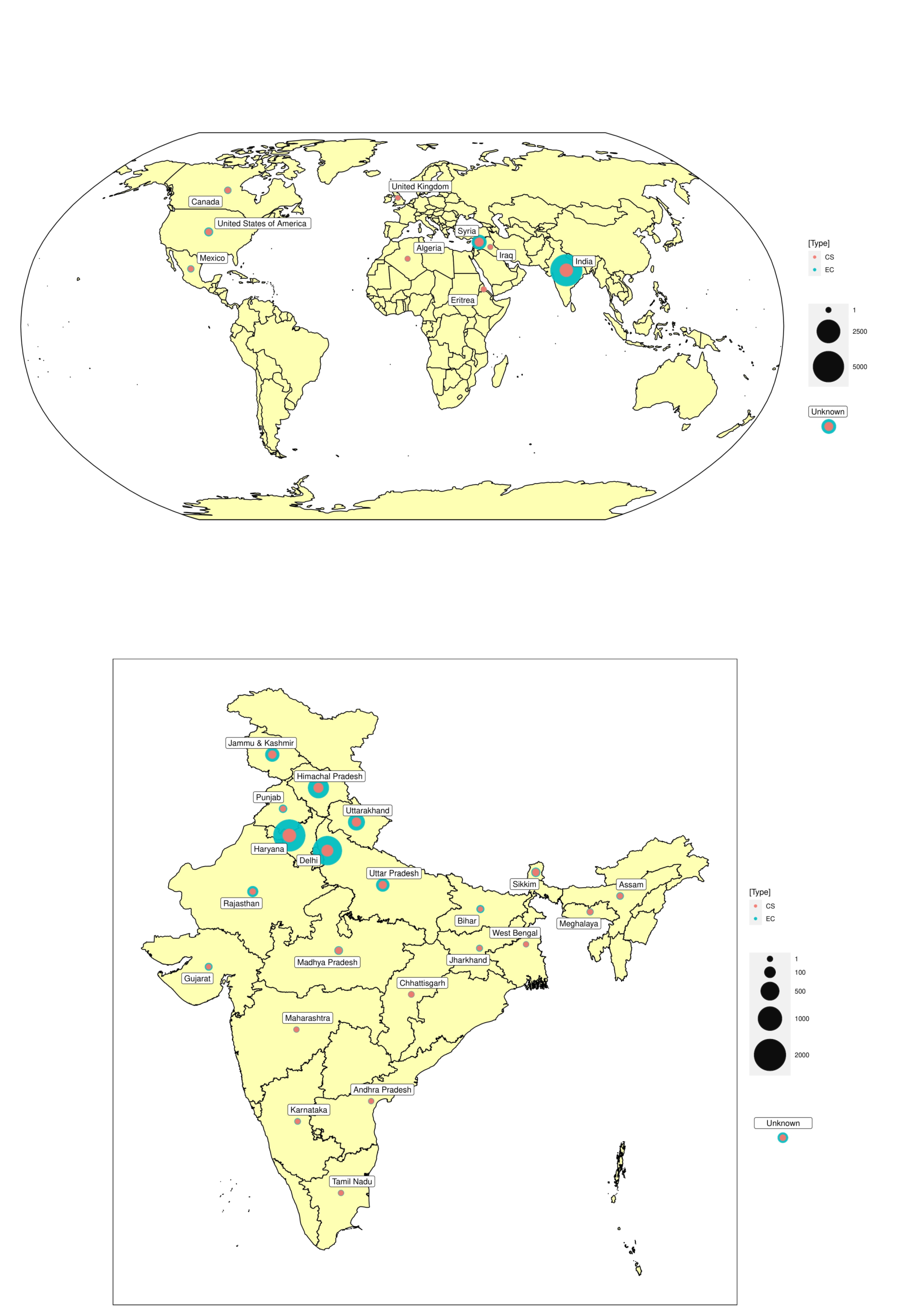

Supplement: Supplementary Figure 6 — Relative geographic representation of collection sites of barley germplasm in the entire collection (EC) and core set (CS). [file Image_6.TIF]

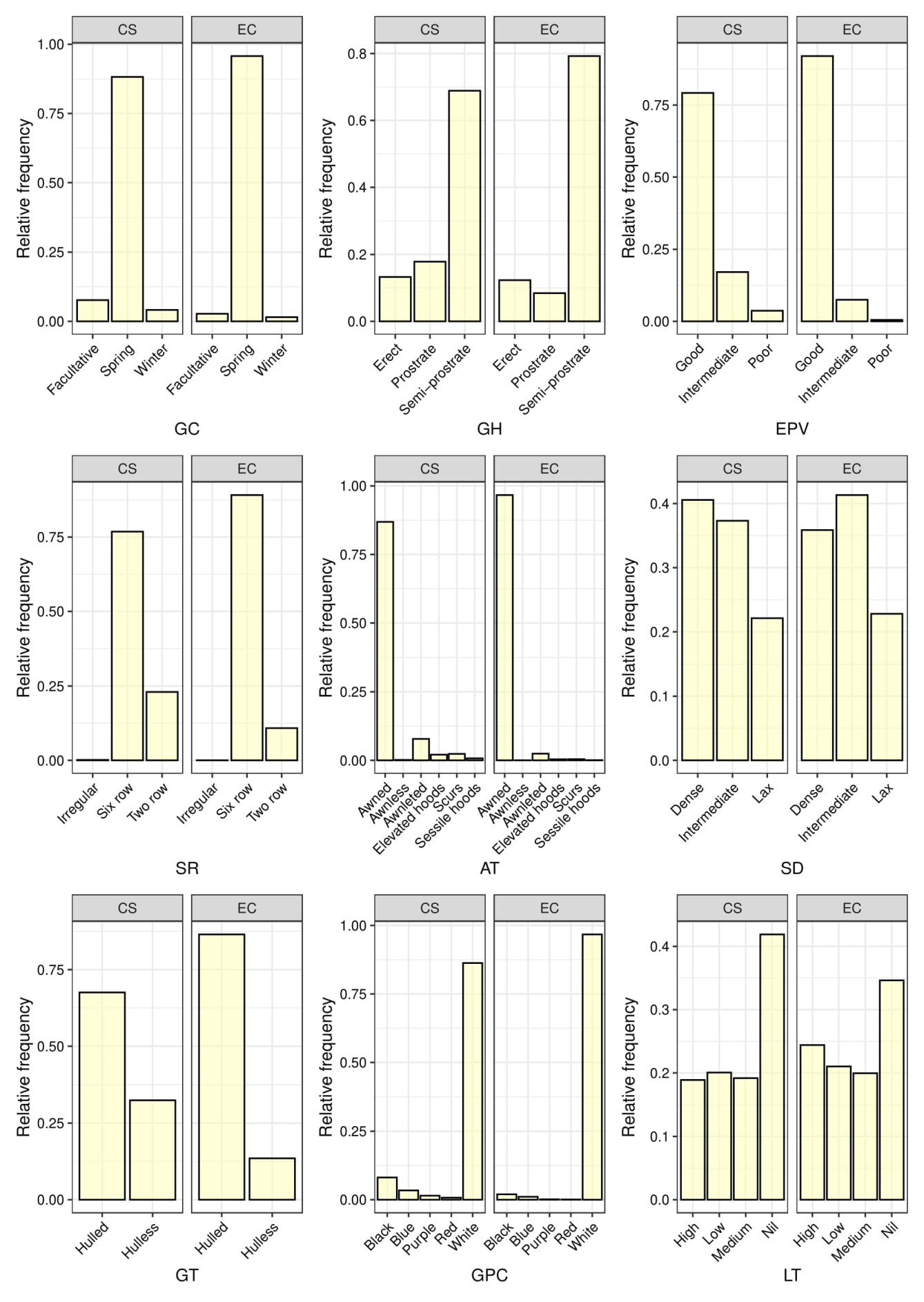

Supplement: Supplementary Figure 7 — Relative frequency bar plots of qualitative traits in the entire collection (EC) and core set (CS). [file Image_7.TIF]
