# Supplementary material for: Phenotypic Characterization, Genetic Diversity Assessment in 6,778 Accessions of Barley (Hordeum vulgare L. ssp. vulgare) Germplasm Conserved in National Genebank of India and Development of a Core Set
Source: Front Plant Sci. 2022 Feb 24;13:771920. doi: 10.3389/fpls.2022.771920 (PMC8913045; doi:10.3389/fpls.2022.771920)
Supplement: Supplementary file 8 [file Table_1.DOCX]

**Supplementary Table 1:** Source/collection areas and biological status of 6,778 barley accessions used in the study

| **Indigenous collection (IC; 5351 accessions)** | | | | | |
| --- | --- | --- | --- | --- | --- |
| **State** | **Total no. of accessions** | **Biological status^#^** | **State** | **Total no. of Accessions** | **Biological status** |
| Andhra Pradesh | 1 | BL | Madhya Pradesh | 27 | LR (5), RC (12), UN (9), GC (1) |
| Assam | 11 | BL (6), EL (1), LR (4) | Maharashtra | 1 | LR |
| Bihar | 17 | LR (16), EL (1) | Meghalaya | 7 | LR (2), UN (5) |
| Chhattisgarh | 3 | LR (2), UN (1) | Punjab | 22 | LR (15), RC (7) |
| Gujarat | 14 | LR | Rajasthan | 85 | RC (16), EL (4), LR (65) |
| Himachal Pradesh | 686 | BL (2), EL (106), LR (179), GS (38), RC (8), UN (353) | Sikkim | 35 | EL (1), LR (34) |
| Jammu and Kashmir | 215 | BL (1), EL (99), LR (33), RC (2), UN (80) | Tamil Nadu | 1 | LR |
| Haryana* | 2002 | BL (845), EL (1100), LR (8), RC (19), GS (30) | Uttar Pradesh | 176 | EL (58), GS (2), LR (100), RC (12), UN (4) |
| Delhi* | 1602 | EL (184), BL (1390), RC (14), TR (10), UN (4) | Uttarakhand | 367 | BL (2), EL (113), LR (161), RC (5), TR (19), UN (67) |
| Jharkhand | 5 | LR | West Bengal | 1 | RC |
| Karnataka | 3 | LR | Unknown | 70 | UN |
| **Exotic collection (EC; 1427 accessions)** | | | | | |
| **Country** | **Total no. of accessions** | **Biological status** | **Country** | **Total no. of Accessions** | **Biological status** |
| Algeria | 1 | UN | Eritrea | 2 | UN |
| Canada | 24 | BL (5), UN (11), LR (8) | USA | 86 | BL (9), EL (9), LR (66), UN (1), TR (1) |
| Mexico | 20 | BL (19), LR (1) | Iraq | 1 | BL |
| Syrian Arab Republic | 664 | BL (93), LR (280), EL (37), UN (254) | Unknown | 628 | UN |
| UK | 1 | BL |  |  |  |

Abbreviations: BL, breeding line; EL, elite line; LR, landrace, RC, released cultivar, GS, genetic stock, WT, wild type; TR, traditional cultivar; UN, unknown

*The higher number of accessions can be attributed to the presence of barley breeding centres which are major donors to National Genebank.

^#^Biological status mentioned here is provided by the donor institute who submitted material to National Genebank.
