# Supplementary material for: Phenotypic Characterization, Genetic Diversity Assessment in 6,778 Accessions of Barley (Hordeum vulgare L. ssp. vulgare) Germplasm Conserved in National Genebank of India and Development of a Core Set
Source: Front Plant Sci. 2022 Feb 24;13:771920. doi: 10.3389/fpls.2022.771920 (PMC8913045; doi:10.3389/fpls.2022.771920)
Supplement: Supplementary file 10 [file Table_3.DOCX]

**Supplementary Table 3:** Correlation coefficients between eight quantitative traits studied in the entire collection (below diagonal) and core set (above diagonal) of barley germplasm

|  | **DPM** | **DSE** | **GNS** | **HGW** | **PH** | **SL** | **STG** | **GY** |
| --- | --- | --- | --- | --- | --- | --- | --- | --- |
| **DPM** |  | 0.69** | 0.05 | -0.06 | 0.31** | 0.09* | 0.21** | 0.17** |
| **DSE** | 0.58** |  | 0.00 | -0.18** | 0.11** | 0.02 | 0.20** | -0.05 |
| **GNS** | 0.04** | -0.01 |  | -0.21** | 0.29** | 0.14** | 0.20** | 0.20** |
| **HGW** | 0.03** | -0.15** | -0.10** |  | 0.12** | 0.23** | 0.17** | 0.29** |
| **PH** | 0.38** | 0.11** | 0.24** | 0.18** |  | 0.23** | 0.28** | 0.52** |
| **SL** | 0.07** | -0.01 | 0.24** | 0.17** | 0.26** |  | 0.50** | 0.24** |
| **STG** | 0.19** | 0.26** | 0.22** | 0.15** | 0.20** | 0.48** |  | 0.22** |
| **GY** | 0.23** | -0.05** | 0.18** | 0.30** | 0.53** | 0.21** | 0.16** |  |

Abbreviations: DSE, days to 75% spike emergence; DPM, days to 80% physiological maturity; PH, plant height (cm); NGS, number of grains per spike; SL, spike length (cm); STG, spikelet triplet groups; HGW, hundred grain weight (g); GY, grain yield per metre row (g)

* and ** indicate significant differences at 5% and 1% probability level, respectively.
